# Potential Role of Polyphenols in Platelet Aggregation and Blood Coagulation

**DOI:** 10.3390/jcdd13050219

**Published:** 2026-05-20

**Authors:** XinYi Wu, Dina Muharib, Christine Boesch, Julia S. Gauer, Robert A. S. Ariëns

**Affiliations:** 1 Leeds Thrombosis Network, Discovery and Translational Science Department, Leeds Institute of Cardiovascular and Metabolic Medicine, University of Leeds, Leeds LS2 9NL, UK; kdtf4577@leeds.ac.uk (X.W.); r.a.s.ariens@leeds.ac.uk (R.A.S.A.); 2 School of Food Science and Nutrition, Faculty of Environment, University of Leeds, Leeds LS2 9NL, UK; c.bosch@leeds.ac.uk

**Keywords:** polyphenols, platelets, fibrinogen, clot structure, blood coagulation, cardiovascular

## Abstract

Cardiovascular diseases (CVDs) are a significant health burden worldwide. One of the key pathological processes underlying CVD is thrombosis–the formation of a blood clot (thrombus) within the blood vessel. Thrombus composition typically includes fibrin, platelets, red blood cells, leukocytes, and neutrophil extracellular traps (NETs). Polyphenols, a diverse group of naturally occurring compounds abundant in plant-based foods, have shown potential cardiovascular protective properties. This review discusses and summarizes the effects of polyphenols on the endothelium, platelet function and activity, and blood coagulation, and how this may potentially contribute to attenuated thrombus formation. The available evidence discussed in this review suggests that polyphenols may confer cardiovascular benefits not only through antioxidant and anti-inflammatory actions, but also by directly modulating thrombosis-related mechanisms. Nevertheless, in vivo studies remain limited, and the lack of standardized procedures contributes to discrepancies among reported results. Moreover, differences in compound structure, absorption and bioavailability should be considered when interpreting findings and their potential application as part of preventative strategies. The evidence presented in this review suggests that polyphenols may offer benefits towards lowering thrombosis risk and reducing recurrence among patients with thrombosis, although additional studies are required to further explore their mechanistic effects.

## 1. Introduction

Cardiovascular diseases (CVD) are a leading cause of global mortality, with increasing prevalence linked to diabetes, hypertension, and obesity [1,2,3]. Common CVDs include coronary artery disease, myocardial infarction, venous thromboembolism and stroke, all of which are caused by thrombosis, or blood clots and thromboemboli blocking the circulation [4]. Both arterial and venous thromboses together incur high rates of mortality and morbidity [5,6]. Lifestyle factors, such as sedentary behavior, smoking, and poor diet, elevate the risk of potentially life-threatening thrombosis. A greater intake of fruits and vegetables has been shown to potentially diminish this risk [7,8,9].

**Figure 1:**
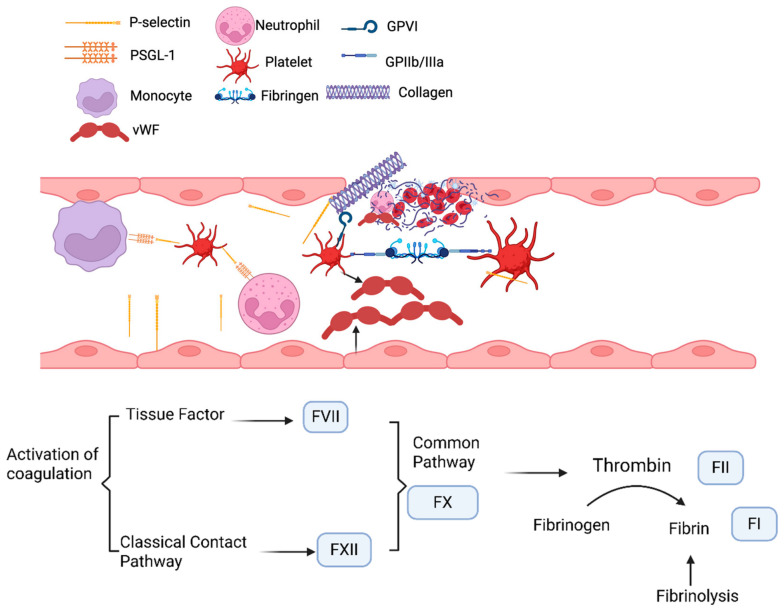
Illustration of the interactions and signaling pathways that drive thrombus formation. Platelets adhere to collagen at sites of vascular injury, a process involving von Willebrand factor (vWF) released from endothelial cells, platelet glycoprotein (GP)VI and other receptors, while fibrinogen binding occurs through the activated GPIIb/IIIa integrin complex. Platelets also bind to monocytes and neutrophils via P-selectin on their surface and PSGL-1 (P-Selectin Glycoprotein Ligand-1) expressed on leukocytes. Activation of GPIIb/IIIa is enhanced by adenosine diphosphate (ADP), which is secreted by activated platelets. The classical contact and tissue factor coagulation pathways converge at Factor (F)X to form the common pathway. This leads to fibrinogen conversion to fibrin (FI) by thrombin (FII), stabilizing the platelet plug and leading to the formation of insoluble clots. Clot dissolution occurs through the process of fibrinolysis.

The thrombus is composed of fibrin, platelets, red blood cells, leukocytes, and neutrophil extracellular traps (NETs) [10]. Following vascular injury, atherosclerotic plaque rupture or local vascular inflammation, platelets undergo activation mediated by exposure to collagen and adhere to the site of damage, subsequently releasing granules containing prothrombotic factors and forming a platelet plug. Concurrent activation of the coagulation cascade results in fibrin formation, which reinforces the platelet plug to form stable clots. Clot dissolution occurs through the enzymatic breakdown of fibrin in the process termed fibrinolysis [11] (Figure 1).

Healthy endothelial cells contribute to thromboresistance by regulating vascular tone, permeability, and the balance between pro- and anti-thrombotic factors [12]. The production of nitric oxide (NO), along with the function of smooth muscle cells, also influences platelet activity and vascular homeostasis, thereby affecting clot formation. Endothelial dysfunction, driven by oxidative stress associated with conditions such as hypertension, diabetes, and hyperlipidemia, creates a pro-thrombotic and pro-inflammatory environment that contributes to the development of atheroprone and prothrombotic vessels [13,14]. The activation of GPIIb/IIIa receptors on platelets enables the binding of fibrinogen, thereby promoting platelet aggregation and the stabilization of the thrombus. Platelet hyperactivity, including increased adhesion and aggregation, and altered activity due to oxidative stress, contributes to a pro-thrombotic phenotype [14,15,16]. Coagulation is initiated via both the contact and tissue factor pathways, which merge at factor X to drive the enzymatic conversion of fibrinogen to fibrin. Denser and lysis-resistant fibrin fiber structures are well characterized risk factors for thrombosis [5,14].

Pharmacological interventions that target platelets or the coagulation pathway activity are commonly prescribed and are effective for thrombosis prevention [17,18,19]. Nevertheless, persistent thrombosis in some high-risk cardiovascular patients, which sometimes can be due to resistance to certain anti-platelet agents (e.g., aspirin), and the increased bleeding risk associated with medications targeting hemostatic mechanisms (anticoagulants) continue to present significant clinical challenges [20,21,22,23].

Dietary habits are known to influence the risk of cardiovascular disease [7,8,9]. Amongst bioactive compounds, plant-derived polyphenols represent one of the most extensively studied classes regarding their potential effects on cardiovascular health and thrombosis risk. The widespread presence of polyphenols in fruits, vegetables, and beverages provides valuable opportunities to explore their potential role in thrombosis prevention. This review will explore the anti-thrombotic effects of polyphenols and discuss the potential of incorporating specific compounds in thrombosis prevention and risk management strategies. Although prior reviews have addressed the antithrombotic properties of bioactive compounds more broadly, this review uniquely integrates evidence on polyphenols, examining their effects on endothelial activity, platelet function, and fibrin clot formation to comprehensively evaluate their potential role in modulating thrombosis. By synthesizing data across experimental models, we highlight evidence that polyphenols may reduce clot formation while addressing the key translational gap between dietary intake and measurable antithrombotic effects.

## 2. Methodology

A systematic search of the Science Scholar database was conducted for studies using the keywords “polyphenol,” “cardiovascular,” “platelet aggregation,” “anti-platelet,” and “thrombus”. In addition, in vivo human studies published between 1995 and 2025 were specifically examined, with a focus on dietary polyphenol intake, particularly through juice-based interventions with clearly defined polyphenol content. These studies were identified using the search terms “polyphenols,” “cardiovascular,” “platelet,” “juice,” and “oral.” Emphasis was placed on investigations evaluating the effects of orally consumed polyphenol-rich products to better reflect clinically relevant dietary exposures. Additional studies were identified by screening the reference lists of relevant articles.

## 3. Polyphenols

Hypertension and type 2 diabetes, both closely linked to lifestyle habits and dietary choices, can significantly increase the risk of thrombosis [9]. A higher intake of fruits and vegetables has been shown to significantly lower this risk [8,9]. Polyphenols, abundant antioxidants in the human diet, are consumed in greater quantities than vitamin C, for example [24]. Their widespread presence in fruits, vegetables, and beverages highlights their potential as candidates for improved cardiovascular disease (CVD) prevention.

### Polyphenol Structure and Subclasses

Polyphenols are broadly classified into four major subclasses: stilbenes, lignans, phenolic acids, and flavonoids [25]. Compounds from different classes have been shown to have diverse effects on health. Resveratrol, an example of the stilbene class, has previously been associated with antioxidant and platelet anti-aggregatory effects [20,26,27]. Resveratrol is naturally found in a variety of plants such as grapes, almonds, beans, blueberries, raspberries, mulberries, and peanuts [25]. Compounds from the largest of the classes, flavonoids, are also amongst the most widely studied classes. A detailed understanding of their structure and classification is crucial for elucidating their biological functions.

**Figure 2:**
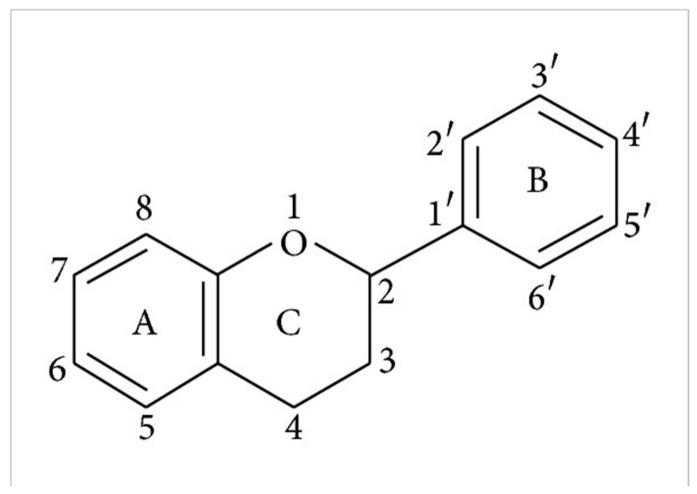
The molecular structure of flavonoids, with the A, B and C aromatic rings. The carbon bone numbers are also shown. Image adapted from [30].

Flavonoid compounds play a critical protective role in plants, particularly in shielding against ultraviolet (UV) radiation, which explains their higher concentrations in the outer layers such as the peel [28]. The concentration and composition of flavonoids can be influenced by various environmental factors, including temperature, UV exposure, seasonal variation, pollution, drought, and salinity stress [27]. Structurally, flavonoids are characterized by two aromatic rings (designated as rings A and B) connected by a three-carbon bridge forming a heterocyclic ring (ring C), which is central to their classification and biological activity (Figure 2) [29]. The degree of saturation of the C2–C3 carbon bond in the C-ring informs the classification of flavonoids into seven subclasses, namely, flavones, flavonols, flavanones, flavanonols, flavanols, anthocyanins and isoflavones [29]. Furthermore, the location and number of functional groups within the flavonoid structure further determine the diverse characteristics of different flavonoid subclasses (Table 1) [29].

The degree of saturation or unsaturation of aromatic rings influences the functional properties of polyphenols. For example, unsaturated flavonoids tend to be more reactive but less stable [29]. This makes them prone to oxidation and interactions with other molecules, and this has been shown to be pivotal in anti-platelet activity, for instance [47]. In addition, the functional groups present in polyphenol compounds of all classes significantly contribute to their biological activities, particularly in terms of antioxidant and anti-inflammatory properties. The presence of hydroxyl (-OH) groups, for example, plays a crucial role in free radical scavenging [29,33,34,47,48,49]. A greater number of -OH groups in flavonoids is typically associated with enhanced antioxidant, anti-platelet and anti-inflammatory effects [29,33,34,47,48,49]. While all polyphenol classes share the core functional group of the reactive hydroxyl group, the unique C6–C3–C6 skeleton of flavonoids creates a more stable and flexible conjugated electron system (Table 1). This structural feature renders their biological activity patterns somewhat consistent across multiple contexts. For example, when ADP was used as the platelet activator, monohydroxylated flavones with a substitution at position 6 of the A-ring (e.g., 6-hydroxyflavone) exhibited relatively higher anti-aggregatory activity, whereas flavonoids hydroxylated at position 7 (e.g., 7-hydroxyflavone) showed comparatively lower potency [50]. Furthermore, methyl groups introduced at double bonds in the structure of polyphenol compounds of all classes increase conformational constraint [50,51]. For instance, resveratrol can be converted to dimethyl resveratrol, which is conformationally more constrained and demonstrated superior suppressive effects on neutrophil extracellular trap (NET) formation than resveratrol itself [50,51].

## 4. Polyphenols and Thrombosis

### 4.1. Vascular System and Endothelial Function

The endothelium is a key player in hemostasis, with endothelial dysfunction leading to increased thrombosis risk [52]. A healthy endothelium supports an optimal coagulation/anticoagulation balance that allows for hemostatic regulation [53]. Flow-mediated dilatation (FMD) of the vessel may also relate to the endothelial function [54]. Previous studies have suggested polyphenols may contribute to vascular tone and endothelial function, and mitigate the crosstalk between endothelial dysfunction and platelet activation by increasing NO availability [55,56]. For instance, CGA (chlorogenic acid), a phenolic acid extracted from coffee, has been shown to benefit vascular health and improve endothelium-dependent vascular reactivity [55]. In the in vivo efficacy study, where coffee was used as the oral intervention, it was found that high-polyphenol coffee which contained about 3–4 times more CGA than low-polyphenol coffee, significantly increased the percentage of FMD compared with the control group [55]. Moreover, a flavanol-rich cocoa drink also showed an acute increase in FMD within 2 h after consumption [56].

Oxidative stress, inflammation, and hyperglycemia are well-established risk factors contributing to the development of atherosclerosis, or plaque deposits along the vessel wall [9]. More specifically, reactive oxygen species (ROS) and oxidized low-density lipoproteins (LDL) activate the inflammatory response. Inflammatory monocyte or lipid-laden foam cells then generate the initial fatty streak which eventually becomes an atherosclerotic plaque [57]. Several compounds have been shown to have a positive impact on cardiovascular health and have potentially important preventative effects on plaque formation [20,29,53,58,59,60]. The antioxidant properties of polyphenols involve scavenging of free radicals, inhibition of ROS generation, upregulation of endogenous antioxidant enzymes, and overall reduction in oxidative stress [47,59,61]. Genistein, a soy-derived isoflavone, has been shown to enhance the activity of key antioxidant enzymes such as superoxide dismutase (SOD) and glutathione peroxidase (GPx) in ovariectomized mice, suggesting its potential therapeutic application in oxidative stress-related cardiovascular conditions [60,62]. In addition, 4 week intake of up to 50 mg/kg/day taxifolin feed to diabetic mice showed increased myocardial antioxidative enzymes SOD and GPx, improved antioxidant capacity, and decreased oxidative stress (determined by decreased levels of malondialdehyde; MDA), indicating that taxifolin may inhibit lipid peroxidation [63]. Furthermore, decreased intracellular ROS production was observed in rat cardiomyoblasts cultured under high-glucose conditions, to model diabetic stress, following treatment with up to 20 μg/mL taxifilin [63]. Hesperetin, a flavanone aglycone, and quercetin, a flavonol, were both shown to activate the Nrf2/heme oxygenase-1 (HO-1) signaling pathway [64,65]. This enzyme plays a crucial role in cellular defense against oxidative stress, indicating the potential of these compounds to attenuate ROS [59,64,65]. Furthermore, resveratrol, quercetin and Epigallocatechin gallate (EGCG) reduced platelet-derived ROS in platelets isolated from healthy volunteers under conditions representative of normoglycemia and acute hyperglycemia (5.6 mM or 25 mM glucose in isolation buffer, respectively) [58]. A similar decrease was observed following hesperetin treatment under normoglycemic conditions only, indicative of a limited efficacy of this compound under hyperglycemic conditions [58].

Certain compounds exert anti-inflammatory effects by increasing NO bioavailability, inhibiting the activation of nuclear factor kappa B (NF-κB), and downregulating the expression of pro-inflammatory cytokines such as tumor Necrosis Factor α (TNF-α) [24,26,66]. EGCG has been reported to induce NO production in endothelial cells, thereby promoting vascular health and offering protective effects against atherosclerosis [13,33]. Polyphenol-rich *Aronia melanocarpa* juice was suggested to improve expression and activation of endothelial NOS (eNOS), leading to increased NO production [67]. Another compound, CGA, reduced inflammation by regulating the expression of pro-inflammatory cytokine levels like TNF-α, interleukin-6 (IL-6), interleukin-8 (IL-8), and interferon gamma [68,69]. This same compound also led to a reduction in inflammation-induced NO production by downregulating inducible NO synthase (iNOS) expression [68,70]. In vivo, CGA treatment (400 mg; equivalent to CGA levels in 2 cups of coffee) showed no significant increase in markers of NO bioavailability measured [71]. Nevertheless, the markers used in the aforementioned study as indicators of overall NO were the sum of circulating nitric oxide species and nitrite [56,71]. These markers, however, cannot differentiate whether NO originated from eNOS [72]. This distinction is critical because the two isoforms have opposing physiological and pathophysiological roles. Namely, eNOS-derived NO is generally protective, promoting vasodilation and endothelial health, whereas sustained iNOS-derived NO is often associated with inflammation, oxidative stress, and tissue injury [73]. In a study using spontaneously hypertensive rats (SHR), oral administration of taxifolin at 20 mg/kg/day significantly increased total NOS activity. However, further analysis revealed that eNOS protein levels remained unchanged, while iNOS expression was markedly upregulated. In that context, the apparent increase in NO bioavailability seemed to reflect a shift toward a pro-inflammatory iNOS [74]. It is possible that CGA treatment may simultaneously reduce NO production by downregulating iNOS while at the same time increasing NO production through upregulation of eNOS. Future studies should therefore include isoform-resolved measurements, such as eNOS-specific activity assays, iNOS protein expression analysis, or the use of selective NOS inhibitors, to confidently attribute observed effects to eNOS or iNOS.

Shear stress also influences NO production and contributes to vessel vasodilation, which contributes to improved blood flow and lowers the risk of platelet activation and thrombus formation [75]. Laminar shear stress enhances endothelial NO production primarily by activating vascular endothelial growth factor 2 and the PI3K–Akt pathway, leading to phosphorylation and activation of eNOS [75]. In contrast, oscillatory shear stress activates NF-κB signaling, promoting the expression of pro-atherogenic and pro-inflammatory genes [75]. Red rice bran extract, containing approximately 225 μg/g taxifolin, may regulate the PI3K/Akt pathway, as indicated by the reduced vasorelaxant response in arterial rings pretreated with the soluble guanylate cyclase inhibitor ODQ and the PI3K inhibitor (Wortmannin) [76]. In addition, treatment with 10 μg/mL taxifolin increased Src phosphorylation without producing a significant effect on eNOS phosphorylation [76]. However, exposure to 20 μg/mL taxifolin significantly enhanced phosphorylation of both Src and eNOS [76]. These findings suggest that 10 μg/mL taxifolin may be insufficient to elicit downstream signaling events, including detectable activation of Akt-dependent pathways. These observations highlight the complexity of in vivo NO production and the resulting challenge of determining appropriate dosages of compounds and extracts in both in vitro and clinical studies.

**Figure 3:**
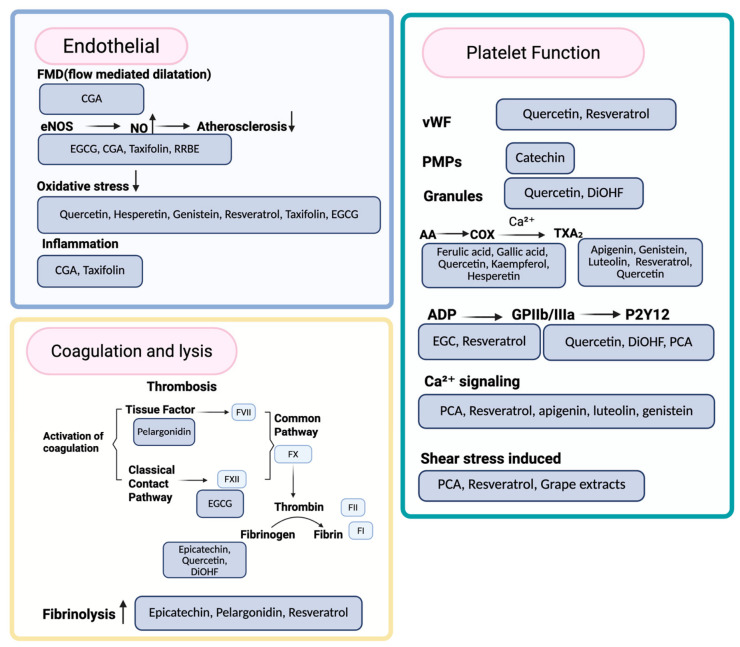
Polyphenols may modulate thrombosis-related pathways by targeting endothelial function, platelet activity, coagulation and fibrinolysis. At the endothelial level, chlorogenic acid (CGA) has been shown to increase percentage flow-mediated dilation (FMD). CGA, epigallocatechin gallate (EGCG), taxifolin and red rice bran extract (RRBE) have been shown to enhance nitric oxide (NO) bioavailability. In addition, studies indicate that compounds such as quercetin, hesperetin, genistein, resveratrol, taxifolin and EGCG reduce oxidative stress, while CGA and taxifolin decrease inflammation, thereby potentially limiting atherogenesis. Certain compounds inhibit platelet activation and aggregation by suppressing ADP–P2Y12 (epigallocatechin (EGC) and resveratrol), GPIIb/IIIa (quercetin and 3′,4′-Dihydroxyflavonol (DiOHF)), Ca^2+^ signaling (protocatechuic acid (PCA), resveratrol, apigenin, luteolin and genistein), and TXA_2_ pathways (ferulic acid, gallic acid, quercetin, kaempferol, hesperetin, apigenin, genistein, luteolin and resveratrol). To add to that, specific compounds also reduce vWF-mediated adhesion (quercetin and resveratrol), platelet microparticles (PMPs; catechin), granule secretion (quercetin and DiOHF), and shear-induced activation (PCA, resveratrol and grape extract). Certain compounds also affect the coagulation and fibrinolysis systems by attenuating thrombin and factor Xa activity (pelargonidin), affecting contact pathway activation (EGCG), modulating fibrin formation (epicatechin, quercetin, and DiOHF), and promoting fibrinolysis (epicatechin, pelargonidin, and resveratrol). Collectively, these effects may contribute to an antithrombotic state.

The effects of polyphenols on mechanisms associated with vascular and endothelium function contributing to thrombosis are summarized in Table 2 and visually represented in Figure 3.

### 4.2. Platelet Function

Platelets are essential mediators of hemostasis, acting through a coordinated sequence of functional responses. These responses are commonly categorized into three principal processes: platelet adhesion, platelet activation, and platelet aggregation [88].

Resveratrol has demonstrated potential to reduce platelet adhesion to collagen (50 µg/mL) under type 2 diabetic conditions [89]. Mechanistically, this may involve modulation of von Willebrand factor (vWF), which plays a central role in platelet adhesion under high shear stress [90,91,92,93]. Shear-induced conformational changes in vWF expose platelet-binding domains, thereby promoting adhesion to subendothelial collagen [85,90,91,92]. In addition, treatment of human umbilical vein endothelial cells (HUVECs) with 100 μg/mL resveratrol has been shown to reduce the secretion of IL-8 and secretion and mRNA expression of vWF [78], suggesting a potential additional vascular mechanism. Protocatechuic acid (PCA) significantly reduced vWF binding up to 1 µM, and a reduction in GPIIb/IIIa activation was observed at 25 µM after 3 min of incubation with washed platelets [85]. To further investigate the potential mechanism of action, vWF binding was assessed in the presence and absence of a GPIIb/IIIa blocker. These experiments suggested that PCA primarily interferes with vWF binding to the GPIb receptor rather than to GPIIb/IIIa [85]. Furthermore, both quercetin and 3′,4′-dihydroxyflavonol (DiOHF) significantly reduced GPIIb/IIIa activation, which was measured by PAC-1 binding in human whole blood following stimulation with adenosine diphosphate (ADP), thrombin receptor-activating peptide (TRAP), and collagen [79]. However, given that these findings are largely derived from in vitro and experimental settings, their translation to in vivo or clinical antithrombotic efficacy remains uncertain. Further investigations are required to better characterize the effects of polyphenols on vWF production and their contribution to platelet activation. Furthermore, platelet microparticles (PMPs) may be produced by platelets in the presence of a strong agonist or with increased shear stress [93]. There are some studies that show polyphenols may also contribute to the inhibition of PMPs formation [81,94]. One such study, using black sorghum extract (BSE) containing catechins and pentahydroxyflavanone-(3 → 4)-catechin-7-O-glucoside, found that it could reduce collagen-induced platelet aggregation and circulatory PMPs in whole blood [81]. However, the study lacked a clear control for distinguishing PMPs from cell fragments or other platelet debris.

Several experimental measures, including metabolic activity, mitochondrial respiration, and ATP release assays, are commonly employed in studies to assess platelet activation and energy status [89,95]. During platelet activation, granule exocytosis further enhances thrombus formation. The α-granules release a variety of adhesion molecules, including P-selectin, which facilitates interactions among platelets, endothelial cells, and leukocytes, thereby contributing to thromboinflammatory responses [90,96]. In addition, dense granules, which contain small molecules such as ADP, adenosine triphosphate (ATP) and calcium ions (Ca^2+^), also contribute to platelet activation and aggregation [90,96]. Quercetin, at a concentration of 1 mM, has been shown to significantly inhibit α-granule exocytosis in platelets stimulated with various agonists, including ADP, AA (arachidonic acid), TRAP, and a combination of adrenaline and collagen [79]. Moreover, DiOHF has been shown to suppress dense granule exocytosis, further indicating that specific compounds may modulate distinct stages of platelet secretion and activation [79].

A key platelet activation pathway involves the synthesis of thromboxane A_2_ (TXA_2_). Upon activation, platelets stimulate the release of AA from membrane phospholipids, which is then metabolized by cyclooxygenase enzymes (COX) and thromboxane synthase to generate TXA_2_, a potent inducer of platelet aggregation and vasoconstriction [97]. Polyphenols, including apigenin, genistein and luteolin have been shown to affect the TXA_2_ pathway, leading to decreased Ca^2+^ mobilization in human washed platelets [80]. Furthermore, a study using isolated platelets from both healthy volunteers and individuals with type 2 diabetes demonstrated that resveratrol significantly reduced TXA_2_ release, with a more pronounced inhibitory effect observed in the diabetic group [89]. Another study using platelet-rich plasma showed that ellagic acid, ferulic acid, gallic acid, quercetin, and kaempferol inhibited collagen- and ADP-induced platelet aggregation [77]. This same study also examined the influence of these compounds on COX-1 and COX-2. Ferulic acid, gallic acid, and quercetin exhibited significant inhibitory effects on COX-1, while ferulic acid, gallic acid, and kaempferol reduced COX-2 activity [77]. Quercetin exhibited negligible inhibition of COX-2, suggesting a selective inhibitory profile toward COX-1. Another study showed that treatment with up to 10 μM hesperetin reduced COX-2 protein and mRNA expression in mouse macrophages [64]. These findings suggest that the inhibition of TXA_2_ production by suppressing COX activity may contribute to the anti-platelet activation effects of polyphenols. However, as highlighted by one study, the role of COX enzymes in thrombosis is complex [98]. Both platelets and endothelial cells express COX-1, but their effects differ. In platelets, COX-1 promotes TXA_2_ synthesis and enhances aggregation, whereas in endothelial cells, COX-1 and COX-2 contribute to the production of prostacyclin (PGI_2_), a vasodilator that inhibits platelet aggregation [99]. Under conditions that elevate oxidative stress, such as platelet activation, diabetes, or exposure to EGCG-induced hydroperoxides, evidence suggests that COX-1 assumes a more important role than COX-2 in generating PGI_2_ [100]. Therefore, inhibition of COX pathways may have dual and context-dependent effects, depending on the cell type and specific vascular environment [101].

The Gq-coupled ADP receptor P2Y1 and the Gi-coupled ADP receptor P2Y12 are associated with Ca^2+^ mobilization and phospholipase C activation, respectively [83]. Mice treated with up to 0.5 g/kg (−)-epigallocatechin (EGC) orally showed reduced ADP-induced platelet aggregation [83]. In addition, resveratrol demonstrated a notable ability to suppress platelet activation and metabolism [89]. Specifically, in isolated platelets, resveratrol treatment (0.25 mmol/L) inhibited platelet ATP release and impaired mitochondrial respiration, effects that were particularly pronounced in patients with diabetes [89]. These findings suggest that resveratrol’s anti-thrombotic effects may, in part, be mediated by its capacity to disrupt the energy metabolism required for platelet activation [89].

Ca^2+^, in dense granules, plays a crucial role in platelet activation and aggregation, as well as in the activation of various coagulation factors in both the contact and tissue factor pathways of fibrin formation [90,102]. A study by Marumo et al. investigated the effects of resveratrol on calcium signaling in platelets using thapsigargin, 1-oleoyl-2-acetylglycerol (OAG) and thrombin as stimulators of Ca^2+^ mobilization [86]. Resveratrol significantly reduced thapsigargin-induced Ca^2+^ entry into platelets and subsequent platelet aggregation at concentrations of 6.25 μM and above, and similarly inhibited thrombin-induced Ca^2+^ entry into and aggregation at concentrations greater than 12.5 μM [86]. These findings indicate that resveratrol can inhibit store-operated Ca^2+^ entry. Apigenin, genistein, luteolin, and quercetin have all been reported to inhibit intracellular Ca^2+^ mobilization, at least in part, through antagonism of the TXA_2_ receptor (TP) [80]. The same study also investigated the effects of quercetin on aspirin-treated platelets, reporting no significant reduction in Ca^2+^ mobilization in U46619 (TXA_2_ analog) and thrombin-activated platelets [80]. Rutin also did not significantly inhibit Ca^2+^ mobilization in either signaling pathway examined [80], indicating functional heterogeneity among different classes of polyphenols. Together, these observations suggest that while multiple polyphenols converge on the regulation of Ca^2+^ signaling, they likely act via distinct molecular targets or pathways.

Epicatechin, at high concentrations (100 μM), was also shown to reduce platelet aggregation induced by various agonists (collagen, ADP, Ca^2+^, thrombin, TXA_2_ analog, and AA) as measured by light transmission aggregometry, suggesting modulation of primary hemostatic processes [82]. The same study showed that epicatechin treatment reduced endogenous thrombin potential in platelet-rich plasma samples. Further studies, including whole-blood research, are needed to identify the most effective concentrations for minimizing platelet aggregation and thrombin activity.

Tail bleeding time is a commonly used technique to measure platelet function and primary hemostasis in mice. Intravenous administration of pelargonidin (up to 5.4 μg/mouse) significantly prolonged tail bleeding time, suggesting an effect on primary hemostasis consistent with an anticoagulant response [84]. Conversely, a study comparing PCA treatment with aspirin and clopidogrel in rats showed that PCA did not prolong bleeding time as conventional anti-platelet therapies did [85]. Nevertheless, increased tail bleeding time was also observed by treatment with EGC delivered orally at up to 0.5 g/kg [83]. Collectively, these studies suggest that polyphenols may differentially modulate primary hemostasis, with effects that may be context-dependent and influenced by concomitant anti-platelet treatment.

Shear stress, particularly under flow conditions, is a critical determinant of platelet adhesion and aggregation. In vascular systems, hemodynamic forces strongly influence platelet–surface and platelet–platelet interactions, thereby modulating both the initiation and stability of thrombus formation. In vitro flow-based assays are valuable tools for modeling thrombus formation in blood vessels and evaluating the antithrombotic potential of various compounds [89]. A study using a cone-plate viscometer to simulate the mechanical forces of blood flow in vitro showed that PCA regulated high shear stress (>10,000/s)–induced platelet aggregation in a concentration-dependent manner [85]. Furthermore, resveratrol has been shown to significantly reduce collagen-mediated thrombus formation under flow conditions in healthy volunteers and individuals with diabetes, as demonstrated in a microfluidic-based in vitro study [58,89]. Similarly, polyphenol-rich grape extracts have been reported to inhibit platelet adhesion to fibrinogen under low shear (300/s; representative of venous systems) conditions, suggesting potential use in preventing thrombus formation in venous or low-flow arterial environments [103]. Nevertheless, there were no data presented on the effect of this compound under high shear (>2000/s; representative of arterial systems) stress conditions, which are often present in stenotic vessels and atherosclerotic plaques.

Beyond their effects on platelets, certain polyphenolic compounds also modulate the activity of other blood cells, such as neutrophils. One study demonstrated that pre-treatment with resveratrol (concentrations ≥3 μM) and catechin significantly reduced phorbol-myristate acetate (PMA)-induced neutrophil extracellular trap (NET) formation in HL-60-derived neutrophils [51]. Resveratrol similarly suppressed NET release following dual stimulation with PMA and the oxidized phospholipid 1-palmitoyl-2-(5-oxovaleroyl) phosphatidylcholine (POVPC), but had no effect when POVPC was used alone [51]. Moreover, a planar analog of catechin (PCat) inhibited calcium ionophore (A23187)-induced NET formation, indicating activity against a protein kinase C-independent NETosis pathway [51]. Since NETs are known to alter thrombus architecture, producing denser clots with smaller pore sizes [104], the ability of polyphenols to attenuate NETosis may represent an additional mechanism by which they reduce thrombotic risk. Further studies are needed to fully characterize the impact of these compounds on neutrophil behavior and NET formation.

All in all, the reported findings suggest a key role for polyphenols in modulating platelet adhesion, platelet activity and aggregation, with a potential protective role for altered activity in conditions such as metabolic syndrome and diabetes. The effects of polyphenols on mechanisms associated with platelet function and behavior contributing to thrombosis are visually represented in Figure 3 and summarized in Table 2.

### 4.3. Coagulation

Coagulation is a complex process that leads to the formation of fibrin fibers that provide the structural backbone for the blood clot. As previously mentioned, thrombus formation involves the interaction of fibrin, platelets, red blood cells, leukocytes, and NETs (Figure 1) [102,104,105]. Fibrin fiber structure is a well-characterized risk factor for thrombosis [106]. Fibrin fiber density and clot permeability are strong indicators of the robustness of clots and their susceptibility to undergo dissolution (fibrinolysis) [107,108].

General clotting assays such as activated partial thromboplastin time (APTT) and prothrombin time (PT), are commonly used to assess the coagulation pathways [11]. A study by Ku et al. reported that 10 μM pelargonidin, a compound in the anthocyanin group within the flavonoid class, significantly prolonged APTT and PT by approximately 30% and 27%, respectively, when added to human plasma in vitro [84]. In addition, oral administration of pelargonidin at 5.4 μg in mice increased the APTT and PT by approximately 25% and 23%, respectively, compared with the control group [85]. This suggests that pelargonidin may inhibit the activity of common pathway factors, thrombin and factor Xa (FXa), in a dose-dependent manner [84]. The same study showed that pelargonidin inhibited FXa activation by interfering with the FVIIa-TF complex, highlighting a potential mechanism by which pelargonidin exerts an anticoagulant effect [84]. A separate study showed that plasma from mice fed with EGC (0.25–1.00 g/kg/day, 10 days treatment), a major catechin found in green tea, also had significantly extended APTT but not PT (with a ~34.6% increase in APTT at 0.25 g), indicating EGC may impact the contact pathway of coagulation only [83]. On the other hand, a recent study performing thromboelastometric analysis on plasma from healthy volunteers incubated with compounds (20 µM) for 20 min found no changes following treatment with EGCG (containing gallic acid ester group), resveratrol, and quercetin [58]. Taken together, these discrepancies highlight the importance of study design including choice of assays and experimental models, while paying particular attention to exposure duration, sampling conditions, and metabolic context, when interpreting results. The effects of polyphenols on mechanisms associated with coagulation are visually represented in Figure 3 and summarized in Table 2.

### 4.4. Fibrinolysis

Improved characterizations of the effects of polyphenols on fibrinolysis have also been previously investigated. In a study using HUVECs, pelargonidin was found to suppress the activation of thrombin and FX produced by endothelial cells stimulated with TNF-α [84]. It also induced a pro-fibrinolytic effect by reducing the ratio of plasminogen activator inhibitor-1 (PAI-1) which inhibits fibrinolysis, and tissue plasminogen activator (tPA), which promotes it, suggesting a potential pro-fibrinolytic effect [84]. Moreover, incubation of platelet-poor plasma with up to 1 μM epicatechin, a flavonoid compound, was shown to increase fibrin clot permeability, promoting sensitivity to tPA, and enhancing breakdown of the clot [82,87]. Another study with low concentrations (1–10 µM) of epicatechin showed positive effects on fibrin clot formation in platelet-poor plasma by increasing maximum absorbance, correlating to thicker fibers and less dense networks, as measured by turbidity assays [82,87]. This observation was supported by electron microscopy images, where thicker fibers and larger pores were observed [87]. There was also a concentration-dependent effect of this compound on fibrin clot permeability, supporting the hypothesis that it causes the formation of a looser fibrin structure that is more prone to undergo fibrinolysis [87]. Nevertheless, treatment of HUVECs with 100 μg/mL resveratrol reduced tPA secretion, intracellular tPA and expression at the mRNA level, suggesting a potential anti-fibrinolytic effect [78]. More studies are needed to shed light on the impact of polyphenols on fibrinolysis and the mechanisms underpinning the effects. The effects of polyphenols on mechanisms associated with fibrinolysis are visually represented in Figure 3 and summarized in Table 2.

## 5. Clinical Relevance and Translational Potential

### 5.1. Polyphenol Absorption, Metabolism and Bioavailability

Despite the widespread presence and relatively higher concentration of polyphenols in everyday diets, their biological impact on molecular and cellular mechanisms of thrombosis remains limited. One potential reason for this is their low bioavailability. For example, after oral ingestion, only about 1–2% of anthocyanins retain their original structure upon reaching the systemic circulation [109]. In addition, most flavonoids are present as glycosides in foods, which must be deglycosylated before being absorbed in the small intestine [29,110]. They are then conjugated in liver cells and enter the bloodstream [111]. However, the rate of deglycosylation is related to the structure and position of the sugar substitution [110]. Flavonoids that are not deglycosylated may be hydrolyzed in the colon and subsequently reabsorbed or eliminated [29,112]. Because aglycone polyphenols are generally more readily absorbed, studies have generally focused on flavanols (not present as glycosides) or compound aglycones. For instance, since the flavanone hesperetin, a deglycosylated form of hesperidin, is more readily absorbed in the human body, it is more often used in research [111].

Polyphenols can also interact with dietary components such as proteins, fats, and polysaccharides, affecting their bioavailability [113], which is an important consideration for in vivo research design. For example, a study on chokeberry juice, containing quercetin, anthocyanins, phenolic acids and CGA, suggested a potential contribution from each to the reduction in blood pressure through increased nitric oxide (NO)-mediated vasodilation, a mechanism that also limits platelet adhesion and activation and thereby lowers thrombotic risk [114,115]. However, the study design may be limited by adherence bias due to inconsistent intake and co-consumption with other foods that may alter polyphenol absorption. Furthermore, single-time point measurements, as was the case in the CGA study, do not account for potential slower or prolonged absorption.

There are several other factors that may impact polyphenol absorption and bioavailability. Food processing and natural variability in polyphenol content across different foods or plant species can significantly impact absorption [41,113]. Interindividual variability, including differences in gut microbiota composition, overall health status, or medical history among volunteers, can significantly influence both the extent and efficiency of polyphenol metabolism and absorption. Furthermore, the presence of polyphenols in the background diet in non-treatment groups may confound the outcomes of dietary intervention trials [56]. Although advances in research have improved our understanding of polyphenol bioavailability, considerable variability persists, which needs to be taken into consideration when standardizing intake recommendations or predicting health outcomes [56].

CGA, which hydrolyzes to caffeic acid and quinic acid, has been shown to contribute to the attenuation of blood pressure and endothelial health in an animal model [55,116]. However, no significant differences between high-polyphenol coffee and low-polyphenol coffee in peripheral systolic or diastolic blood pressure were observed in vivo [55]. Several factors may explain this outcome, including prolonged exposure to air during processing or preparation with hot water, which could alter polyphenol stability and influence absorption. Furthermore, polyphenols may not act through immediate mechanisms, as indicated by the biphasic peaks observed in FMD, which likely reflect absorption occurring in the small intestine and later in the colon [55,112]. All in all, the structure, uptake and bioavailability of compounds may all influence the data obtained by studies investigating the effects of polyphenols on thrombus formation and are important considerations for data interpretation and study design.

### 5.2. Important Considerations for Clinical Implementation

As indicated in the bioavailability section above and shown in Table 3, compounds rarely reach plasma levels in their parent form, with metabolites being mostly present in plasma [117,118,119,120,121,122,123,124,125,126,127,128,129]. Plasma levels of parent compounds are usually found at concentrations of less than 100 ng/mL, with resveratrol shown to be present at the highest concentration of ~117–538.3 ng/mL [130], which may indicate a reason for the use of this compound in several studies.

Bioavailability and metabolism are, therefore, critical considerations when determining the dosage of compounds or extracts for human studies. Nevertheless, there are methods available that may improve the bioavailability of polyphenols in the human body. For example, co-administration of the black pepper compound piperine with resveratrol in murine studies increased the maximum serum concentration of resveratrol while delaying the formation of its primary glucuronide metabolite [124]. In addition, liposome system co-delivery of compounds may also be a promising strategy to improve stability and bioavailability of polyphenols [68,131]. To enhance anthocyanin bioavailability specifically, it has been shown that anthocyanins can spontaneously bind to proteins within a food matrix [132]. For example, soy-blueberry complexes formed by combining blueberry juice with soy protein increased total bioaccessibility from 26.3% (control: blueberry juice alone) to 36% [131].

Interestingly, a study showed that following 28 days of supplementation with 1.0 g/day quercetin, plasma quercetin concentrations increased significantly from 0.10 ± 0.09 to 1.5 ± 0.3 μmol/L, indicating that orally administered quercetin is effectively absorbed and detectable in circulation [133]. However, this increase was not accompanied by significant changes in platelet aggregation, thromboxane B_2_ production, lipid profile, or fatty acid composition [133]. These findings challenge the initial assumption that the limited efficacy of dietary polyphenols is primarily due to poor bioavailability and failure to reach systemic circulation. Instead, it suggests that the mere presence of compounds in plasma, even at elevated concentrations, does not necessarily translate into measurable functional effects.

This lack of observed effect may be partially explained by several aspects of the study design. For instance, the relatively small sample size and inclusion of only healthy participants may have limited statistical power and introduced a ceiling effect, as baseline platelet function and cardiovascular markers were already within normal ranges. Moreover, the composition of the supplement, which included additional bioflavonoids and other components, may have introduced potential interactions affecting absorption or activity. Dietary intake was not tightly controlled, particularly with respect to background fat and polyphenol intake, which may have also introduced variability in both baseline platelet responsiveness and compound metabolism. Finally, chronic supplementation over 28 days may have led to physiological adaptation, potentially attenuating observable responses.

Both acute and chronic consumption of polyphenol-rich foods appear to influence hemostatic function, albeit through different mechanisms. For example, the acute consumption of anthocyanin-rich sour cherry demonstrated significant reductions in platelet aggregation within 3–5 h [134]. Nevertheless, the absence of a control group, small sample size, and short observation period reduce the robustness of these findings [134]. In another study, supplementation with a polyphenol-rich extract from *Pinus massoniana* (containing catechins and proanthocyanidins) over 12 weeks in healthy older adults resulted in significant reductions in fibrinogen and oxidative stress markers (i.e., MDA) [135]. Direct measurements of platelet function were not included in this study, limiting conclusions regarding potential antiplatelet activity. The absence of acute time point measurements (e.g., several hours after initial intake) may further limit the ability to detect transient effects associated with peak plasma concentrations. In another study, acute intake of an anthocyanin-rich blackcurrant beverage demonstrated significant inhibitory effects on platelet aggregation in healthy individuals under tightly controlled postprandial conditions. In this randomized, double-blind, crossover trial, participants consumed a standardized high-fat, low-flavonoid meal alongside the intervention, and platelet function was assessed at multiple time points (2 and 4 h) using both ADP (10 and 100 μM) and collagen (0.5 and 1 μg/mL) as agonists [136]. This design enabled the detection of transient, time-dependent reductions in platelet aggregation, with significant treatment effects observed across multiple agonists [136]. Importantly, the study also incorporated rigorous dietary control prior to testing, including a low-flavonoid diet and standardized meals [136], thereby minimizing background variability in polyphenol exposure. Mechanistically, the observed effects appear to be linked not only to the parent anthocyanins per se, which were largely undetectable in plasma, but rather to a spectrum of circulating phenolic metabolites that peaked within 2–4 h post-consumption [136]. Beyond differences in exposure timing, this study also highlights the importance of matrix complexity and metabolic context. The beverage delivered a mixture of anthocyanins and procyanidins alongside sugars and organic acids [136], within a tightly controlled dietary framework, which may have contributed to enhanced absorption kinetics and influenced postprandial physiology. Furthermore, the combination of a high-fat test meal with a low-polyphenol background diet may have amplified the postprandial window of platelet activation, providing a more sensitive physiological context in which inhibitory effects could be observed, in contrast to fasting conditions used in other studies. To add to that, the use of multiple agonists and repeated measurements increased sensitivity to detect functional changes. In another study, supplementation with 2 g/day grape polyphenols during a 31-day overfeeding intervention in males was shown to modulate adipose tissue gene expression, particularly pathways related to angiogenesis and mitochondrial metabolism. In addition, polyphenol intake attenuated platelet endothelial cell adhesion molecule-1 (CD31) at the protein level [137]. Taken together, these findings suggest that the anti-platelet effects of dietary polyphenols are highly context-dependent, with acute, postprandial conditions, metabolite profiles, and experimental sensitivity playing critical roles.

As well as bioavailability of compounds, other important considerations include potential toxicity and adverse effects. For instance, gastrointestinal adverse effects, including diarrhea, nausea, and abdominal pain, were reported by at least one of the 28 participants who consumed doses greater than 1 g of resveratrol administered as uncoated immediate-release tablets [138].

Another important consideration for human interventions is the potential effects of compounds or extracts in the presence of thrombosis management medication. For instance, a study suggested that resveratrol may provide benefit in patients with aspirin resistance by reducing platelet aggregation [20]. However, the experimental design was limited to a comparison between aspirin-resistant patients with and without resveratrol supplementation, without inclusion of alternative therapeutic strategies [20]. Given that multiple anti-platelet and anticoagulant agents (e.g., clopidogrel) could be used in the management of aspirin resistance and thrombotic risk [139], the absence of such comparative groups limits the ability to determine the relative efficacy and clinical relevance of resveratrol treatment. Incorporating established anti-platelet and anticoagulation treatments into future study designs would enable a more robust evaluation of resveratrol’s potential therapeutic value. In rats, sweet almond oil exhibited intrinsic anti-angiogenic activity, inhibiting aortic growth at concentrations up to 50 µg/mL. When used as an adjuvant to aspirin, it further demonstrated modulatory potential by significantly reducing vessel growth compared with the control group [140]. The reported in vivo effects of polyphenols on thrombosis-related mechanisms are summarized in Table 4.

Overall, while current evidence is preliminary and methodologically limited, these findings collectively indicate potential clinical relevance and translational potential of including polyphenolic compounds as part of thrombosis management and prevention strategies. Nevertheless, further well-controlled, comparative clinical studies that ensure gender equality and inclusive representation of different ethnicities and patient groups are required to confirm efficacy and safety.

## 6. Conclusions

Polyphenols represent a diverse group of bioactive compounds found abundantly in plant-based foods. The biological activity and absorption profiles of different compounds vary considerably, likely due to differences in their chemical structures and dietary sources.

Polyphenols have been shown to reduce CVD risk by attenuating oxidative stress, suppressing inflammatory responses, and modulating thrombotic pathways. Certain compounds have been shown to enhance susceptibility to fibrinolysis, while others inhibit the generation of coagulation factors such as thrombin and FXa and prolong clotting times. Therefore, different types of polyphenols may modulate distinct pathways related to thrombus formation, thereby influencing thrombosis risk through varying mechanisms. While in vitro studies have elucidated multiple mechanisms through which polyphenols may contribute to improved endothelial health and vascular function and exert antiplatelet and anticoagulation effects, human studies have produced inconsistent findings, likely influenced by factors such as low bioavailability.

The available evidence discussed in this review indicates that polyphenols could show promise as complementary dietary agents that may help reduce thrombosis risk, offering benefits beyond those provided by existing therapies targeting coagulation, platelet function, lipid levels, and hypertension. Nevertheless, limited bioavailability, use of differing models and samples in research, and the multifactorial, pathway-spanning actions of polyphenols may underlie inconsistencies within reported effects. Further research is warranted to clarify the in vivo efficacy of polyphenols and to identify effective strategies to improve their systemic bioavailability. Moreover, targeted investigations involving patients with specific cardiovascular conditions are also required. With ongoing advances in analytical and experimental technologies, future research may provide deeper insights into the mechanistic role of polyphenols in thrombosis modulation and support their potential for clinical translation.

## Figures and Tables

**Table 1:** Flavonoid subclasses and the degree of saturation of the C2–C3 in the C ring, functional group (source from: [31,32]), food source and representative compounds.

Subclass of Flavonoid	Saturation Degree	Presence of Hydroxyl Group	Structure	Representative Compounds	Example Food Sources
Flavones	C2=C3 (unsaturated)	No	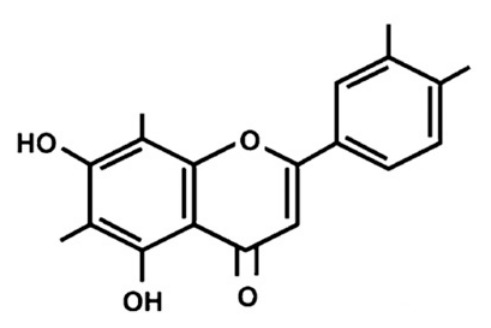	Luteolin Apigenin	beans, wheat sprouts, celery and tea [32,33,34]
Flavonols	C2=C3 (unsaturated)	Yes	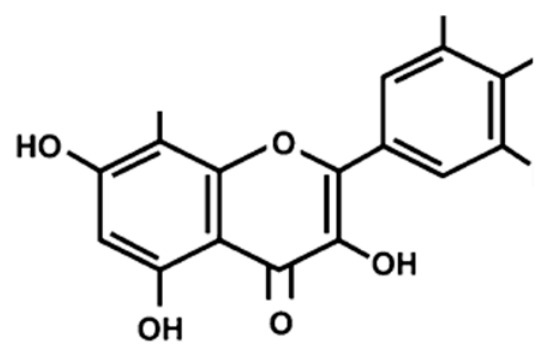	Quercetin Kaempferol	green tea, black tea, red wine, lentils, and broad beans [30,32,33,34,35]
Flavanone	C2-C3 (saturated)	No	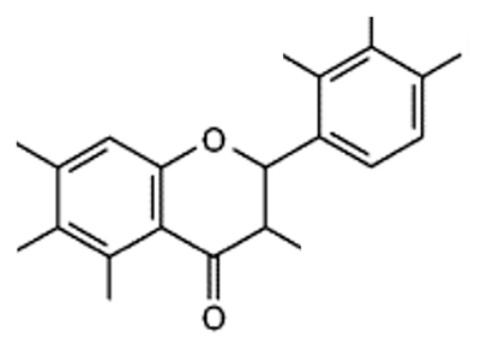	Hesperetin Naringenin Rutin [33,34]	oranges, tangerine, lemons, cocoa [33,34,36]
Flavanonols	C2–C3 (saturated)	Yes	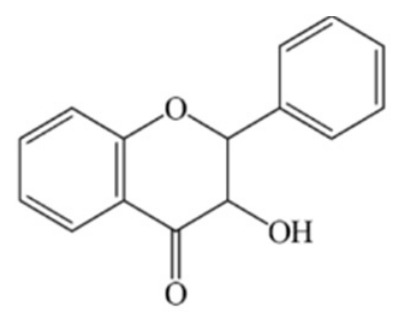	Taxifolin	milk thistle, siberian larch [37,38]
Flavanols	C2–C3 (saturated)	Yes	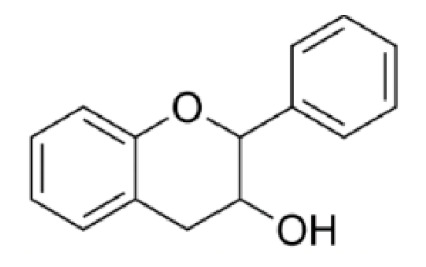	Catechins Epicatechin	apples, peaches [32]
Anthocyanins	C2=C3 (unsaturated)	Yes	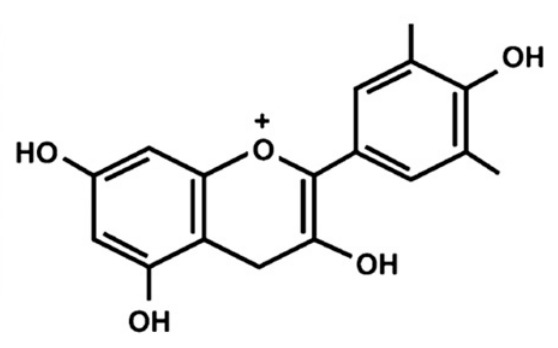	Cyanidin Petunidin Peonidin Pelargonidin [39]	red wine, blackberry, grape, cabbage, tomatoes, eggplant [30,33,34,40,41,42]
Isoflavones	C2=C3 (unsaturated)	No	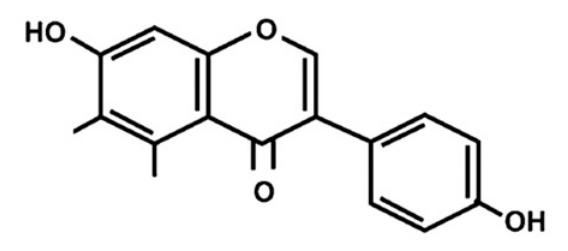	Genistein Daidzein Glycitein [43,44]	Soybeans, green split peas, chickpeas, black beans, lima beans, clover sprouts, and sunflower seeds [33,45,46]

**Table 2:** Summary of reported effects of polyphenols on the endothelium, platelet activity, coagulation and fibrinolysis.

Compound	Model and Dosage	Reported Effects	Reference
**Endothelium**			
Genistein	Female mice fed 0.13–1.3 mg/day for 14 days	↑ SOD (low doses: 0.13–0.26 mg) ↑ GPx (all doses)	[62]
Hesperetin	murine macrophages + LPS-induced inflammation treated with 10–40 μM	↓ TNF-α, IL-6, IL-1β (up to 10 μM) ↓ iNOS and COX-2 expression (up to 10 μM) ↓ NF-κB activation (up to 10 μM) ↑ HO-1 expression ↑ Nrf2 activation	[64]
Quercetin	human L02 hepatocytes and HepG2 cells treated with 7.5–30 μM	↓ ROS and MDA ↑ Antioxidant defenses ↑ Nrf2 and HO-1 expression	[65]
	Platelet-poor plasma from healthy volunteers treated with 0–100 μM	↓ COX-2	[77]
Red Rice Bran Extract (RRBE)	Porcine coronary artery rings with endothelium treated with 1–100 μg/mL	↓ intracellular ROS (up to 20 μg/mL) ↑ phosphorylation of Src (up to 10 μg/mL) and eNOS	[76]
Resveratrol	HUVECs treated with 25–100 μg/mL for 24–48 h	↓ vWF and IL-8 secretion	[78]
Taxifolin	STZ-induced diabetic mice fed 25–100 mg/kg/day for 4 weeks	↓ myocardial MDA (up to 25 mg/kg) ↑ SOD (up to 50 mg/kg)	[63]
	Isolated abdominal aorta from spontaneously hypertensive rats treated with 20 mg/kg/day	↑ Total NOS activity ↑ iNOS protein ↓ COX-2 protein	[74]
**Platelets**			
3′,4′-Dihydroxyflavonol (DiOHF)	Human washed platelets and platelet-rich plasma treated with 0.1–1.0 mM	↓ platelet aggregation (collagen, ADP, AA) ↓ ATP release from dense granules (collagen, AA) ↓ thrombin induced dense granule exocytosis. ↓ GPIIb/IIIa activation	[79]
Apigenin	Human washed platelets treated with 10–100 μM	↓ Ca^2+^ mobilization	[80]
Catechin derivative (black sorghum extract; BSE)	Whole blood from healthy volunteers treated with 5–40 μg/mL	↓ Collagen-induced platelet aggregation. ↓ Circulatory PMPs	[81]
Epicatechin	Platelet-rich plasma from healthy volunteers treated with 1–100 μM	↓ platelet aggregation ↓ ETP	[82]
Epigallocatechin (EGC)	Platelet-rich plasma from mice exposed to 0.25–1.0 g/kg/day	↓ platelet aggregation	[83]
Epigallocatechin gallate (EGCG)	Healthy volunteer washed platelets treated with 20 μM in normo- and acute hyperglycemia	↓ platelet ROS (normo- and hyperglycemia) ↓ mitochondrial density (normoglycemia) ↓ Ca^2+^ flux (hyperglycemia)	[58]
Ellagic acid	Platelet-rich plasma from healthy volunteers treated with 25–700 μM	↑ platelet aggregation (collagen, ADP) ↓ COX-1 and COX-2	[77]
Ferulic acid			
Gallic acid			
Genistein	Human washed platelets treated with 10–100 μM	↓ Ca^2+^ mobilization	[62]
Hesperetin	Healthy volunteer washed platelets treated with 20 μM in normo- and acute hyperglycemia	↓ platelet ROS (normoglycemia)	[58]
Kaempferol	Platelet-rich plasma from healthy volunteers treated with 25–700 μM	↑ platelet aggregation (collagen, ADP) ↓ COX-1 and COX-2	[77]
Luteolin	Human washed platelets treated with 10–100 μM	↓ Ca^2+^ mobilization	[80]
Pelargonidin	Wild-type mice administered a 5.4–16.3 μg dose	↓ platelet aggregation ↑ bleeding time	[84]
Protocatechuic acid (PCA)	Human washed platelets and platelet-rich plasma treated with 1–50 μM	↓ platelet activation by shear stress ↓ intracellular Ca^2+^ mobilization ↓ α-granule and dense granule secretion ↓ GPIIb/IIIa activation ↓ vWF binding to platelets	[85]
	Platelet-rich plasma from rats administered with 5–100 mg/kg single dose	↓ thrombus formation	
Quercetin	Human washed platelets treated with 10–100 μM	↓ Ca^2+^ mobilization	[80]
	Human washed platelets and platelet-rich plasma treated with 0.1–1.0 mM	↓ platelet aggregation (collagen, ADP, AA) ↓ ATP release from dense granules (collagen, AA) ↓ Thrombin-induced dense granule exocytosis ↓ GPIIb/IIIa activation ↓ α granule exocytosis	[79]
	Healthy volunteer washed platelets treated with 20 μM in normo- and acute hyperglycemia	↓ platelet ROS (normo- and hyperglycemia) ↓ mitochondrial density (normoglycemia)	[58]
	Platelet-rich plasma from healthy volunteer treated with 25–700 μM	↑ antiplatelet activity (collagen, ADP)	[77]
Resveratrol	Isolated platelets from healthy and type 2 diabetes individuals treated with 0.25 mmol/L	↓ platelet adhesion to collagen (healthy and diabetes) ↓ TXA_2_ production (healthy and diabetes) ↓ collagen-induced platelet aggregation (diabetes)	[78]
	Human washed platelets treated with 3–50 μM	↓ Platelet aggregation (thrombin, thapsigargin) ↓ Ca^2+^ entry (thrombin, thapsigargin)	[86]
	Healthy volunteer washed platelets treated with 20 μM in normo- and acute hyperglycemia	↓ platelet ROS (normo- and hyperglycemia) ↓ procoagulant platelet number (normo- and hyperglycemia) ↓ basal and maximal respiration (normo- and hyperglycemia) ↓ Ca^2+^ flux (hyperglycemia) ↓ platelet deposition on collagen (normoglycemia)	[58]
	Platelets from high-risk cardiovascular patients on aspirin treated with 10 μM	↓ platelet aggregation in ASA-R (collagen, epinephrine)	[20]
**Coagulation and Fibrinolysis**			
Epicatechin	Platelet-poor plasma from healthy volunteers treated with 1–100 μM	↓ clot lysis time	[82]
		↑ clot permeability ↑ turbidity maximum absorbance	[87]
Epigallocatechin (EGC)	Platelet-rich plasma from mice exposed to 0.25–1.0 g/kg/day	↑ APTT ↑ tail bleeding time	[83]
Pelargonidin	Human plasma and HUVECs treated with 1–100 μM	↑ APTT and PT ↓ TNF-α induced PAI 1 secretion. ↓ PAI 1/t-PA ratio ↓ fibrin polymerization	[84]
Resveratrol	HUVECs treated with 25–100 μg/mL for 24–48 h	↓ t-PA secretion and expression	[78]

↑ represents an increase, ↓ represents a decrease. SOD: superoxide dismutase. GPx: glutathione peroxidase. TNF-α: Tumor Necrosis Factor alpha. IL-6: Interleukin-6. IL-1β: Interleukin-1 beta. LPS: lipopolysaccharide. NF-κB: Nuclear factor kappa-light-chain-enhancer of activated B cells. iNOS: Inducible NO synthase, inducible nitric oxide synthase. eNOS: Endothelial nitric oxide synthase. HO-1: Heme oxygenase-1. COX: cyclooxygenase enzymes. TXA_2_: thromboxane A_2_. TRAP: thrombin receptor-activating peptide. ADP: adenosine diphosphate. ATP: adenosine triphosphate. Ca^2+^: calcium ions. AA: arachidonic acid. Nrf2: Nuclear factor erythroid 2-related factor 2. MDA: Malondialdehyde. STZ: streptozotocin. vWF: von Willebrand factor. PMPs: platelet microparticles. ETP: Endogenous Thrombin Potential. ROS: reactive oxygen species. ASA-R: Aspirin resistance. PAI-1: plasminogen activator inhibitor-1. tPA: tissue plasminogen activator. APTT: Activated partial thromboplastin time (intrinsic pathway). PT: Prothrombin time (extrinsic pathway).

**Table 3:** Bioavailability parameters of representative compounds of the 4 polyphenolic classes.

Representative Compound (Class)	Typical Dietary Intake (mg/day)	Major Circulating Forms	Peak Plasma Concentration	Time to Peak Plasma Concentration (h)	References
Quercetin (flavonoid)	5–40	Quercetin-3′-O-sulfate Quercetin-3-O-glucuronide 3′-methylquercetin-3-glucuronide	~28–427 ng/mL	0.6–2.5	[117,118,119,120,121,122]
chlorogenic acid (CGA; phenolic acid)	500–1000	3-caffeoylquinic, 4-caffeoylquinic 5-caffeoylquinic acids dicaffeoylquinic acids	~7.66 ± 2.5 μmol/L	0.5–4.0	[121,122]
Resveratrol (stilbene)	0.8	Resveratrol-3-O-β-glucuronide Resveratrol-3-O-sulfate Dihydroresveratrol Cis- and trans-resveratrol	parent: ~117–538.3 ng/mL metabolites: ~3248.4 ng/mL	1–6	[123,124,125,126,127,130]
7-Hydroxymaitairesinol (7-HMR; lignans)	1–72	Enterolactone (ENL) Enterodiol	~4.8 ± 1.64 ng/mL	1	[128,129]

**Table 4:** Reported in vivo effects of polyphenols on thrombosis-related mechanisms.

Compound/Extract	Model and Dosage	Reported Effects	Reference
Pine bark extract (*Pinus massoniana*)	432 mg pine bark extract for 12 weeks; 60 healthy volunteers	↓ MDA ↑ IL-6	[135]
Sour cherry (fresh fruit) Anthocyanins	250 g sour cherry for 5 h; 6 volunteers	↓ A6 (AA) ↓MS (AA) ↓ AUC (AA)	[134]
Grape polyphenols supplementation	2 g/day (250 mg/capsule, 8 capsules/day) for 31 days; 42 healthy males	modulation of angiogenesis genes (e.g GATA2, PPARγ, and HIF2α) ↓ PECAM1/CD31	[137]
Blackcurrant (*Ribes nigrum*) beverage	200 mL beverage for 24 h; 23 healthy volunteers	↓FMD ↓ IL-8 ↓ platelet aggregation (ADP, collagen)	[136]
Quercetin	4 capsules/day for 28 days; for 27 healthy volunteers	↑ plasma quercetin concentration - no change in platelet aggregation (collagen) and TXA_2_ production	[133]
chlorogenic acid (CGA)	LPC (89 mg CGA + 110 mg caffeine) and HPC (310 mg CGA + 110 mg caffeine) for 5 h; 15 healthy males	↑ total plasma CGA metabolite ↑ FMD in HPC and LPC group	[55]
	450–900 mg 5-CQA for 4 h; 15 healthy males	↑ Total plasma CGA metabolite - no change in FMD	[55]
(−)-Epicatechin	200 mg for 4 h; 15 healthy males	↑ FMD	[55]

↑ represents an increase, ↓ represents a decrease, and - represents no significant change. MDA: Malondialdehyde. IL-6: Interleukin-6. A6: (amplitude of platelet aggregation). MS: Maximal slope of platelet aggregation. AUC: area under the curve of platelet aggregation. FMD: Flow-mediated dilatation. IL-8: Interleukin-8.LPC: low polyphenol coffee. HPC: high polyphenol coffee. TXA_2_: thromboxane A_2_. PECAM1/CD31: platelet and endothelial cell adhesion molecule. ADP: adenosine diphosphate. AA: arachidonic acid. 5-CQA: 5-caffeoylquinic acid, the main CGA in coffee.

## Data Availability

No new data were created or analyzed in this study.
